# Invasive Plants as a Source of Polyphenols with High Radical Scavenging Activity

**DOI:** 10.3390/plants14030467

**Published:** 2025-02-05

**Authors:** Oskars Purmalis, Linards Klavins, Evelina Niedrite, Marcis Mezulis, Maris Klavins

**Affiliations:** Department of Environmental Science, University of Latvia, LV-1004 Riga, Latvia

**Keywords:** invasive plants, *Lupinus polyphyllus*, *Impatiens glandulifera*, *Heracleum sosnowskyi*, *Solidago canadensis*, *Echinocystis lobata*, *Elodea canadensis*, polyphenols, antiradical activity, valorisation, plant biomass, bioeconomy

## Abstract

The wide occurrence and expansive nature of invasive plant species are worldwide problems because these plants by their competitive character can lead to the loss of biodiversity. As a result, they significantly disrupt ecosystems, create economic damage and threats to human health, and diminish the quality of recreational resources. Therefore, sustainable, bio-based solutions are needed for their control, focusing on the utilization of their biomass after eradication. To better understand the potential application possibilities of invasive plants and their potential role in bioeconomy, species such as *Lupinus polyphyllus*—Lindl., *Impatiens glandulifera* Royle, *Heracleum sosnowskyi* Manden, *Solidago canadensis* L., *Echinocystis lobata* (Michx.), and *Elodea canadensis* Michx. were studied. These plants are not only widely spread but also form dense mono-stands and produce substantial amounts of biomass, which provides more options for their harvesting. In particular, their composition was analysed to assess the feasibility of their use for bioactive compound extraction. The amount of total polyphenols and flavonoids was determined in various parts of the studied invasive plants, and their corresponding radical scavenging activities were determined using DPPH, ABTS, FRAP and CUPRAC. The studied invasive plants are rich sources of polyphenols, and the highest concentrations were found in *Impatiens glandulifera* leaves, reaching a concentration of 7.78–11.75 g GAE/100 g DW, but in *Lupinus polyphyllus*, the highest concentrations of polyphenols were identified in the extracts of the flowers (12.77 g GAE/100 g DW) and leaves (11.88 g GAE/100 g DW) of the plant. Among the various plant parts studied, the leaves and flowers consistently showed the highest concentrations of polyphenols and flavonoids, as well as the greatest antioxidant and radical scavenging activities. These findings underscore the potential of invasive plant biomass as a source of valuable bioactive substances, particularly polyphenols, after the eradication of these invasive species.

## 1. Introduction

Invasive alien species and their spreading are among the major drivers of biodiversity loss and alteration of ecosystem functionality [1,2,3]. Although all Member States of the European Union (EU) have adopted Regulation 1143/2014 [4] on the prevention and management of the introduction and spread of invasive, non-native species, the ongoing expansion of invasive plants is still evident. Globalization [5], the magnitude of human activities [6,7,8,9], and global warming [2,3] are the main drivers of invasive species spreading, which causes estimated economic losses [1,2] in the EU of up to 116 billion EUR annually [10]. From over 1500 species introduced and spreading around the Earth [11], the list of invasive plants with major concern has reached 41 species in Europe [12], while the Nature Conservation Agency in Latvia has listed 36 species [6]. The most common invasive plant species in Latvia and Northern Europe are Sosnowsky’s hogweed (*Heracleum sosnowskyi*), Himalayan balsam (*Impatiens glandulifera*), and Canadian goldenrod (*Solidago canadensis*), while other species like garden lupin (*Lupinus polyphyllus*), wild cucumber (*Echinocystis lobata*), and waterweed (*Elodea canadensis*) are emerging in significant habitats across the country [13,14].

Despite significant financial resources and efforts spent on controlling biological invasions [1], it is still important to estimate the effectiveness of different existing and newly developed eradication methods [15,16,17,18,19,20], study the phytochemical composition of invasive plants to better understand their competitiveness in the environment, and foster various applications of produced plant biomass. Lately, a shift from research into eradication [21,22] to usage of biomass [23,24,25,26] and its valorisation [27,28,29] has been observed as plant biomass can be a valuable resource for the bioeconomy. Valorisation methods for plant biomass can be grouped by type of application: composting [30,31,32]; bioenergy [22,28,33,34]; food supplements [28,35]; therapeutic [21,24]; biologically active substances [36,37,38,39]; and other specific applications [38].

The potential of invasive plants for valorisation depends on the composition of their primary and secondary metabolites: cellulose, hemicellulose, fibres, fatty acids, polysaccharides, phenolics, vitamins, essential oils, lipids, and phytohormones [40,41,42]. Among phytochemicals, phenolic compounds have already shown their health-beneficial potential in different types of biomass [43]. Polyphenols are bioactive compounds of phytochemical origin, secondary metabolites found in various types of plants, vegetables, herbs, and fruits, as well as in their secondary products [44,45]. These compounds consist of various phenolics such as simple phenols, benzoic and cinnamic acid derivatives, coumarins, tannins, lignins, lignans, and flavonoids [45]. Polyphenols are synthesized in plants, and they play different specific roles in the survival of plants, mostly as a response factor to ecological and physiological stress (UV radiation, pathogens, insects, wounds, etc.) [45,46]. For now, over 10,000 phenolic compounds with plant origins have been reported, of which approximately 6500 are flavonoids and 1000 hydrolysable tannins [47]. The abundance of extractable phenolic compounds has attracted the attention of research and development for possible applications, and in vitro studies show that plant polyphenols can also affect various processes in mammalian cells—for example, polyphenols can function as anticarcinogenic and antiatherogenic (inhibiting the formation of atherosclerosis) substances and others [44]. Polyphenols protect the body from free radicals by activating the endogenous immune system and modulating cellular processes [48,49]. It has been observed that polyphenols also work as mutagenicity-reducing substances in human cells [43,45]. Polyphenols have been shown to play a key role in the hypotensive effect of various plant extracts [50].

Although polyphenols have various benefits, the use of invasive plants as their source should be carefully evaluated, considering the overall chemical composition of the biomass and other groups of compounds present. *Heracleum sosnowskyi* is well known for its capability to affect the skin due to the presence of furocoumarins [51], which, when exposed to direct UV light, can cause allergic reactions, skin burns, and dermatitis [52,53]. For *Lupinus polyphyllus*, one of their controlling measures is grazing, despite the presence of toxic alkaloids [54], which limits their potential usage, especially if valuable compounds can be extracted together with alkaloids. Studies show that *Lupinus* spp. plants can contain more than 200 naturally occurring quinolizidine alkaloids [55], but not all alkaloids are characterized as toxic. Quinolizidine alkaloids found in lupines have oral toxicity due to neurological effects [56] and can be synthesized in noticeable amounts within the plant [57]. Furthermore, detailed phytochemical studies should be carried out before wider usage of invasive species phytochemicals, because, for example, seeds can contain cyanogenic glycosides [58], which can limit valorisation options, although it is not widely reported. Despite potential risks, plants and their specific parts still can be used for various applications, and comparison with other species as feed for earthworms has not demonstrated a negative influence [59]. Risk assessment of human health has not been considered for *Impatiens glandulifera* and *Echinocystis lobata*, because of their traditional usage as a food source [60] and other applications for health measures [61].

Concerning the necessity to better understand invasive plant composition and the potential use of their biomass, the aim of this study was to characterize the total polyphenolic and flavonoid content and antiradical, antioxidant activity (DPPH, ABTS, FRAP, CUPRAC) of extracts from six invasive plant species found in Latvia. *Lupinus polyphyllus*, *Impatiens glandulifera*, *Solidago canadensis*, *Heracleum sosnowskyi*, *Elodea canadensis*, and *Echinocystis lobata* are not only widely spread invasive species but also frequently make stands rather than separate individuals, improving possibilities for gathering their biomass. Compositional analysis of these plants provides an addition to existing knowledge by describing the phenolic content in plants located in Northern Europe, therefore indicating potential differences in comparison to territories where these plants are native. Moreover, for plant biomass usage for bioenergy, typically the whole plant is used, leading to another aim of the study, which is to describe the composition of polyphenols and flavonoids in different parts of the plants, which can foster the development of more precisely aimed biotechnologies and applications of plant extracts in the bioeconomy.

## 2. Results

The total content of polyphenols from six different invasive plants (*Lupinus polyphyllus*, *Impatiens glandulifera*, *Solidago canadensis*, *Heracleum sosnowskyi*, *Elodea canadensis*, and *Echinocystis lobata*) and their parts were determined using three different solvents (methanol, ethanol (70%), and isopropanol).

Although methanol is an effective solvent for the extraction of phenolic compounds [49], due to its toxicity other alcoholic solvents also should be considered because of their different properties and potential extraction efficiency. For industrial applications, use of ethanol as a “green” solvent is preferable. With respect to the development of valorisation methods with specifically targeted applications, the collected plants were divided into individual parts, which were used for extraction and analysis.

Total extraction yield, using methanol, ethanol, and isopropanol as extraction solvents, was determined (Table 1). Since different plants and their parts were extracted, the obtained results represent significant variability (ANOVA, *p* < 0.01). As the most effective solvents, methanol and ethanol were identified, while isopropanol showed generally lower extraction efficiency. Due to individual differences between plants and their parts, the amounts of extractable compounds also differed. Leaves and flowers were among the plant parts with the highest measured extraction yields. Isopropanol gave the lowest amount of extraction yield from the roots and fibrous stems of the studied plants; in contrast, ethanol and methanol did not show such a pattern (Table 1).

### 2.1. Polyphenols

**Figure 1:**
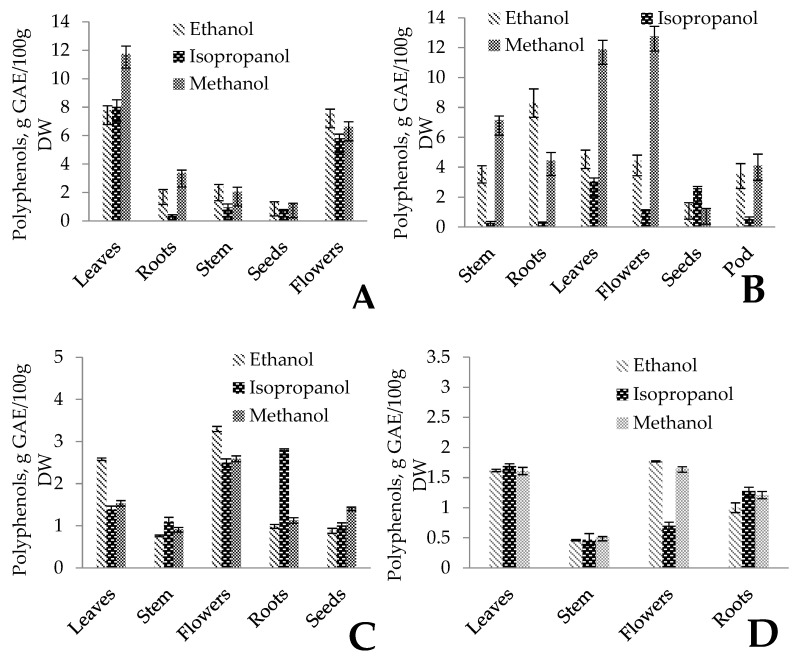
The total amount of polyphenols in the parts of *Impatiens glandulifera* (**A**), *Lupinus polyphyllus* (**B**), *Heracleum sosnowskyi* (**C**), and *Solidago canadensis* (**D**). Error bars represent the standard deviation of measurements (*n* = 5).

Total polyphenolic content was expressed as g of gallic acid equivalent by dry weight (DW) (g GAE/100 g DW). The results for *Impatiens glandulifera* demonstrated the highest values of polyphenolic contents in extracts from leaves and flowers, while seeds had the lowest amount of polyphenols (Figure 1A). For leaves, the total content of polyphenols in the methanol extracts reached 11.75 g GAE/100 g DW, while in ethanol there was up to 7.78 and up to 7.55 g GAE/100 g DW in flowers. Since polyphenols are secondary metabolites vital for plants against ecological and physiological stress, these concentrations can be influenced by environmental conditions in sampling plots and may vary between plants growing in different climatic conditions. Applied solvents have no significant differences in extraction efficiency, although methanol tends to have a higher yield of phenolic compounds, especially in leaves of *Impatiens glandulifera* (ANOVA, *p* > 0.05).

In the case of *Lupinus polyphyllus*, the highest concentrations of polyphenols were identified in the methanol extracts of flowers (12.77 g GAE/100 g DW) and leaves (11.88 g GAE/100 g DW), and ethanol extracts of roots (8.34 g GAE/100 g DW) (Figure 1B). Similarly to *Impatiens glandulifera* and seeds of *Lupinus polyphyllus*, a low amount of polyphenols was found, which can be affected not only by the plant’s physiological shift to the autumn season but also by a higher content of lipids in the seeds [60,62,63]. The extraction efficiency of *Lupinus polyphyllus* indicated much higher variability than that of *Impatiens glandulifera*, but similarly, the lowest concentration of polyphenolics was found in isopropanol extracts.

**Figure 2:**
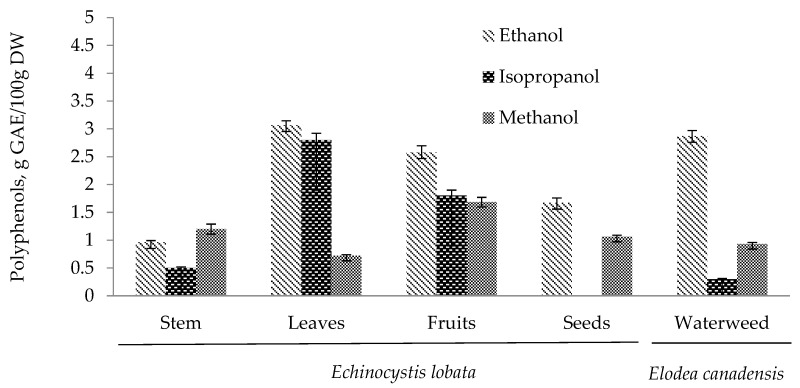
The total amount of polyphenols in the parts of *Echinocystis lobata* and *Elodea canadensis* (waterweed). Error bars represent the standard deviation of measurements (*n* = 5).

In the case of *Echinocystis lobata*, the content of polyphenols was analysed for the above-ground parts of the plant—stem, leaves, fruit, and seeds—because of the poorly developed root system of the plant which can reduce possible valorisation options. The highest concentration of total polyphenols was determined for the ethanol leaf extract, reaching 3.06 g GAE/100 g DW (Figure 2). Although the achieved result is comparable with the results of Hosakatte et al. [60], other parts of plants and solvents showed lower concentrations of polyphenols. Significant variation between parts of plants of different phenolic compounds in *Echinocystis lobata* has a noticeable impact on the results achieved by different solvents (ANOVA, *p* < 0.05). These differences can be observed for isopropanol extracts, where no polyphenols were found in extracts from seeds and lower concentrations for the stem. Similar patterns of concentrations and their differences between solvents were also found in *Elodea canadensis* (waterweed) (Figure 2). These major differences can be explained by higher polarity (containing more OH groups) of polyphenols in these parts of *Echinocystis lobata* and *Elodea canadensis.*

From the plants studied, *Heracleum sosnowskyi* poses serious threats to ecosystems due to its invasive character. It is among the highest concern species in Europe and also can produce biomass in noticeable amounts; therefore, it has the potential to be used for different valorisation options [64]. Total phenolic contents in *H. sosnowskyi* were lower than in *Impatiens glandulifera* and *Lupinus polyphyllus* and varied from 0.76 g GAE/100 g DW in ethanol extracts of the stem to 3.30 g GAE/100 g DW in ethanol extracts of flowers (Figure 1C). Another noticeable difference with other plants is the proportionally lower yield of polyphenols depending on the solvents used for extraction from *Solidago canadensis*. In comparison to the other studied plants, *Heracleum sosnowskyi* contains significantly more essential oils [65], which not only may affect the content of phenolic compounds but also may affect the extraction process. Also, for *Solidago canadensis* the parts richest in polyphenols, similar to the other studied invasive plants, were the leaves and flowers, reaching up to 1.77 g GAE/100 g DW in ethanol extract (Figure 1D). Although the total amount of polyphenols for *Solidago canadensis* was one of the lowest among the studied plants, this is not the main criterion with which to evaluate the valorisation potential of plants, because despite the low polyphenol amounts achieved, the antioxidant activity in *Solidago canadensis* was noticeably higher than in the other studied species. Concentration differences of polyphenols and flavonoids in plants and their parts can be affected also by the location of stands and existing climatic conditions, since polyphenols are secondary metabolites with different specific roles in the survival of plants.

### 2.2. Flavonoids

The total flavonoid content in the invasive plants was analysed (Table 2). The obtained results showed lower total flavonoid concentrations in the seeds of the studied plants for all of the used extraction solvents.

Isopropanol showed the lowest efficiency of flavonoid extraction, giving results below the limit of detection for several species and their parts. Flavonoid concentrations were less variable and lower than polyphenols but still represented a remarkable proportion compared to the total amount of polyphenols, especially for *Heracleum sosnowskyi*, *Solidago canadensis*, and *Echinocystis lobata*. The results represent individual differences between plants and their parts without a trackable pattern, indicating the necessity for precisely targeted valorisation options, especially when focusing on a particular part of the plant and specific species.

### 2.3. Radical Scavenging Activity of Invasive Plant Extracts

Since the studied invasive plants contain polyphenols and flavonoids in amounts that can be suitable for the development of applications and valorisation, their antiradical and antioxidant activity were assessed using several methods, such as DPPH, ABTS, FRAP, and CUPRAC. The potential of invasive plants for use as food supplements and other applications where antioxidant activity is an important parameter can be therefore evaluated.

Although a strong correlation between the total polyphenol concentration and antiradical activity was expected, the results of the estimated antiradical activity show a different pattern (Table 3). The highest antiradical activity was demonstrated by the parts of *Impatiens glandulifera*, reaching 335.85 for the ethanol extract, 348.28 for isopropanol, and 343.06 mg Trolox equivalent/100 g DW for the methanol extract of the flowers. A similar pattern of DPPH was shown by the results for the stem extracts (257.03–279.12 mg Trolox equivalent/100 g DW), although the highest values of total polyphenols were observed for the flowers and leaves of *Impatiens glandulifera*. The flowers and leaves of all the studied plants had higher detected antiradical activity, and *Impatiens glandulifera*, *Heracleum sosnowskyi*, and *Solidago canadensis* had significantly higher activity than other studied plants (ANOVA, *p* < 0.01). Noticeably higher concentrations were demonstrated by the roots of *Impatiens glandulifera* (172.11–220.74 mg Trolox equivalent/100 g DW) and *Heracleum sosnowskyi* (151.78–183.46 mg Trolox equivalent/100 g DW), as well as the stem of *Impatiens glandulifera* despite the lower amount of total polyphenols and flavonoids. On the contrary for *Lupinus polyphyllus*, with one of the highest content of polyphenols, detected antiradical activity was rather low, reaching 73.56–120.06 mg Trolox equivalent/100 g DW for flowers and 66.85–105.3 mg Trolox equivalent/100 g DW for leaves. The seeds of the analysed plants have a very low content of polyphenols and also low antiradical activity. Therefore, seeds that are rich in fatty acids have other potential valorisation options and also applications. The least effective studied species for antiradical activity were *Echinocystis lobata* and *Elodea canadensis*. With respect to solvents used, the variety of plants and their parts did not show clear patterns indicating differences in plant phytochemical composition, but overall ethanol extracts showed higher antiradical activity in more cases, while the lowest values were observed for the isopropanol extracts.

The ferric-reducing antioxidant power (FRAP) assay is widely used for characterising foods and biological fluids to assess the content of labile antioxidants [66]. Our results (Table 4) show that, although FRAP varied significantly between plants and their parts, the differences were not viable for simple statistical comparison (ANOVA, *p* < 0.05). Still, a recognizable correlation between FRAP and polyphenols could be detected, showing higher FRAP for plant parts with higher amounts of polyphenols and flavonoids. The plants with higher FRAP activity were *Solidago canadensis*, *Heracleum sosnowskyi*, and *Impatiens glandulifera.* The stems of the studied plants typically had lower FRAP values and the majority of seeds. These results only partially can be explained by the lower total amount of extracted substances and concentrations of polyphenols and flavonoids from these parts. Significantly lower FRAP values were determined for isopropanol extracts as well as the proportion of differences in comparison to other solvents and to other detected antioxidant activities (ANOVA, *p* < 0.05). The FRAP analysis methodology differs from other detected biological activities because of its acidic testing environment, thus directly influencing the binding capacity with iron using isopropanol. In the case of seeds, this effect is magnified because of the presence of fatty acids and the tendency to precipitation in solutions describing determined lower values using isopropanol.

The ABTS method for antioxidant capacity measurements was also used to determine the antioxidant potential in samples with higher concentrations of lipophilic substances. The achieved results indicate that the highest ABTS values were recorded for extracts from flowers and roots (Table 5). Although the activity measured by ABTS was comparably low, it was proportional to other parts of the plants. From the perspective of valorisation and extraction after the eradication of invasive plants in the flowering period without the ability to separate individual plant parts, the high antiradical activity of flowers can be reduced by proportionally a much higher total volume of leaves and stems in biomass. In such an approach (mass ratio between flowers and above-ground plant parts), the highest values can be achieved using *Impatiens glandulifera* and *Lupinus polyphyllus* for extraction, while *Echinocystis lobata* provides the lowest radical scavenging values. The solvents used demonstrated significantly lower differences in the ABTS values than other analysed antiradical and antioxidant activities (ANOVA, *p* < 0.01). Marginally higher values were reached when ethanol was used for the extraction of *Heracleum sosnowskyi* and *Impatiens glandulifera*, while methanol extracts showed higher values for parts of *Solidago canadensis*, *Lupinus polyphyllus*, and *Elodea canadensis*.

The cupric reducing antioxidant capacity (CUPRAC) method showed significant variability not only between plants but also between different parts of the plants. The studied plants and their parts provided noticeable data dispersion which limits the direct comparison of polyphenol and flavonoid content to the dataset. CUPRAC values (Table 6) were significantly higher for flowers, reaching up to 1.947 in *Impatiens glandulifera* and 5.074 g TE/100 g DW in *Heracleum sosnowskyi*’s ethanol extracts (ANOVA, *p* < 0.001). Although much lower than flowers, the leaves and roots also demonstrated viable antioxidant capacity. Overall, *Impatiens glandulifera* parts had lower CUPARC values, while *Lupinus polyphyllus* and *Heracleum sosnowskyi* had higher values, and that pattern only partially corresponds to the total amount of polyphenols. This indicates that the dominant role of polyphenols corresponds to antiradical activity; however, other substances potentially present in the extracts can also affect the antioxidant potential, for example, ascorbic acid. Evaluating the obtained results for different solvents, in general, ethanol extracts had slightly higher values, but isopropanol showed comparably lower results.

### 2.4. Alkaloid Contents of Different L. polyphyllus Parts

**Figure 3:**
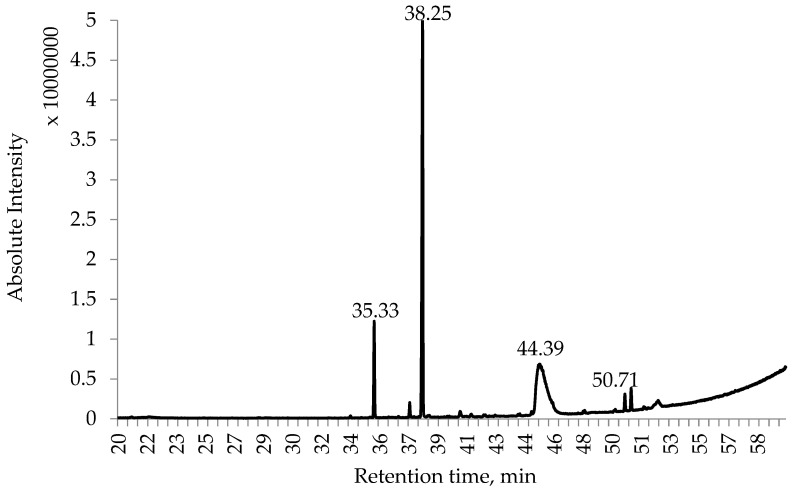
A representative chromatogram of the found alkaloids in *Lupinus polyphyllus* ethanol extracts of seeds. The numbers above peaks represent the retention time (RT) of the identified compounds in Table 7 and Table 8.

Lupines are known to contain different alkaloids, and their contents may vary depending on the species [67]. In this study, the invasive plant *L. polyphyllus* was investigated for its alkaloid contents not only in the whole plant (in the total biomass) but in each part of the plant separately. The highest concentration of alkaloids was found in the ethanol extracts prepared from *L. polyphyllus* seeds (up to 3.9 g/100 g DW) (Table 7). Also, the leaves contained significant amounts of alkaloids (up to 2.4 g/100 g DW) (Table 8), while the roots had the lowest alkaloid concentration found in all of the plant parts studied (as low as 96 mg/100 g DW). The obtained extracts contained relatively few compounds that could be identified directly using GC/MS—only a few peaks (Figure 3) corresponding to typical quinolizidine-type alkaloid mass spectra could be seen; however, it is clear that the extracts also contained other substances, for example, triglycerides, fatty acids, alkanes, etc., that are a natural part of the *L. polyphyllus* biomass.

The studied *L. polyphyllus* parts were believed to have the same alkaloid composition; however, our results showed that some of the analysed parts contained a higher number of quinolizidine-type alkaloids than others. Tetrahydrobromifoline and 13-tigloyloxylupanine, which is the main lupin alkaloid, were found in all the studied parts (Table 7 and Table 8). D-lupanine was found in all the parts except flowers and leaves (Table 7 and Table 8). These results show that, depending on the alkaloids of interest, specific parts of this plant could be used for extraction. Such an approach, in terms of biorefinery, would simplify the purification and fractionation approaches to be used to obtain these substances for further investigation. Moreover, considering the solvents used and the amount of each specific alkaloid extracted using them, the extraction process can be manipulated and adjusted according to needs. The most efficient of the solvents, depending on the extraction needs, are either aqueous ethanol or methanol, while isopropanol showed no specificity towards the extraction of alkaloids of interest. This could be explained by the different polarities of the used solvents—aqueous ethanol, being the most polar, showed the highest extraction yield, followed by methanol, and finally, the least polar, isopropanol, had the lowest alkaloid extraction yields. These results also indicate that the possibility of using even more diluted ethanol exists, which could be more attractive for large-scale extraction. It must be noted that optimisation and the concentration of the used solvents should be optimised to achieve, possibly, even higher extraction yields, and this should preferably be carried out by the creation of a statistically reliable experimental design backed by a mathematical optimisation approach (Box–Behnken response surface methodology).

## 3. Discussion

Invasive alien species and their invasions are a significant threat to ecosystems worldwide. The presence of invasive species can be influenced by numerous factors, and one of the pathways is the allelopathic nature of these plants. One of their success strategies is the production of chemicals that can affect the growth of other species, which can be studied altogether with overall plant phytochemical composition for the development of valorisation perspectives of their biomass after eradication [37]. Although invasive plants and their biomass are not widely used materials, the biomass itself can be considered an attractive source from which biofuels, biochemicals [68], and other value-added products, including applications as a resource for bioeconomy solutions, can be produced. Our focus was to analyse biologically active substances (polyphenols, flavonoids) in six widely spread invasive plant species, with the focus not only on the whole plant but also on individual parts of the plant.

The results of total extraction yield of methanol, ethanol, and isopropanol extracts from parts of studied plants demonstrate significant extractable proportions with the highest yield for most polar solvent—ethanol. This aspect has an impact on the solubility of different substances, for example, fatty acids [69], carbohydrates, oils, lipids [70], flavonoids, and polyphenols [50]. Isopropanol had a significantly lower extraction yield, particularly from the stems of plants, with the exception of *Lupinus polyphyllus*. Still, the treatment with alcoholic solvents leaves a high proportion of unextractable parts of plants, which can be further processed or used by other biomass utilization approaches.

Polyphenols are one of the most biologically active groups of compounds synthesized in plants as secondary metabolites, and they play different specific roles in the survival of plants, protecting them from biotic and abiotic stress factors [43]. Therefore, the amounts found in specific parts of plants can differ due to abiotic factors and geography [28]. The obtained amount of polyphenols in the analysed samples of plants and their parts were different, with the highest concentrations in the flowers and leaves of *Impatiens glandulifera* followed by *Lupinus polyphyllus.* Although the results of the total amount of polyphenols are comparable with other studies [71] of *Impatiens* spp. above-ground plant biomass, the analysis of leaves and flowers in our study demonstrated significantly higher concentrations. However, our results for the stem (0.95–2.42 g GAE/100 g DW) are comparable to the achieved results in [71] (0.88–2.16 g GAE/100 g DW), indicating determined differences for individual parts which depend on proportionally higher amounts of stems in total plant biomass (ANOVA, *p* < 0.01), thus indicating the benefits of specific plant part usage for extraction to increase the yield of phytochemicals of main interest. This approach was also used in analysing the roots of *Lupinus polyphyllus* as a source of phenolic compounds [72], where such dominant phenolic compounds as gallic acid, catechin, epigallocatechin, ellagic acid, epicatechin, catechin gallate, and epicatechin gallate were determined. The leaves were the richest part of the plants, based on the concentration of polyphenols, as in other studies [73,74] of *Heracleum* spp. However, our study demonstrated that flowers have slightly higher concentrations, although the results were comparable and similar for leaves. Despite lower values of polyphenols in *Heracleum sosnowskyi* in comparison to *Impatiens glandulifera* and *Lupinus polyphyllus*, other studies approved *Heracleum* spp., which is a source rich in a variety of phytochemical compounds that are recognized as valuable [75] for different applications despite the presence of furanocoumarins [76] and their ability to affect human skin. Noticeable similarities to *Heracleum sosnowskyi* in polyphenol concentrations were observed for *Solidago canadensis* and *Echinocystis lobata*, and in these plants the richest parts were the leaves and flowers. Other studies [77] achieved results reaching only 0.117–0.38 g GAE/100 g DW for *Solidago canadensis*, but in that case, the plants were extracted with 50% (*v*/*v*) aqueous ethanol, which may have affected the extractable amount, because a study by Radusiene et al. [78] achieved results similar to our study. Previously *E. canadensis* was not considered a significant source of polyphenols, with more attention given to its potential as a protein source [79]. However, various applications of these plants have been studied due to their substantial biomass production. One such application is the extraction of flavonoids, where the primary flavonoids identified include apigenin, luteolin, and chrysoeriol 7-O-diglucuronides [80], which were later studied for their allelopathic activity [81]. Our results demonstrate a comparable total polyphenol and flavonoid content to other studied plants, such as *Heracleum sosnowskyi*, *Solidago canadensis*, and *Echinocystis lobata*.

The total amount of polyphenols differs between plant species and their parts, as well as depending on the extraction solvent used. The abundance of plant-derived polyphenols [47] and the specificity required for their detection has led to only a few studies which have tried to characterize the dominant polyphenols. For example, in *Echinocystis lobata* most polyphenols consist of three major compounds: isoquercitin, rutin, and quercitin with lesser amounts of kaempferol, p-coumaric acid, and ferulic acid [82]. In *Heracleum* spp. chlorogenic acid is the dominant compound followed by caffeic acid, apigenin, and kaempferol [73]. These compounds influence not only their allelopathic properties [83], but also antioxidant activity. In *Impatiens* spp., hyperoside is the dominant phenolic compound, with isoquercitin, trifolin, and nicotiflorin also identified [71]. Similarly, the dominant phenolic compounds in *Lupinus polyphyllus* include gallic acid, catechin, epigallocatechin, ellagic acid, epicatechin, catechin gallate, and epicatechin gallate [72]. In *Solidago canadensis*, the most abundant polyphenols were chlorogenic acid, rutin [77], and quercitrin [78]. The presence of dominant polyphenols such as chlorogenic acid in *Heracleum* spp. [73] and *Solidago canadensis* resulted in lower variability in polyphenol extraction efficiency across solvents. In contrast, other plants showed higher fluctuations in polyphenol content depending on the solvent used. Significant variations in the phenolic compound profiles of *Echinocystis lobata*, *Elodea canadensis*, and *Lupinus polyphyllus* were observed with different solvents, particularly isopropanol extracts. These differences can be attributed to the higher polarity (due to the presence of more hydroxyl groups) of polyphenols in certain parts of *Echinocystis lobata*, *Lupinus polyphyllus*, and *Elodea canadensis*, as well as the presence of more lipophilic compounds [84], which significantly impact the extraction results of seeds and *Elodea canadensis.*

The lower concentration of flavonoids in plants, particularly invasive plants, reflects a gap in current knowledge. In our study, the total flavonoid content was relatively low, except in flowers, where flavonoids constituted a significant proportion of the total polyphenols. In stems, flavonoids were present in concentration, close to the limit of detection, while in seeds the presence of fatty acids and lipids reduced their overall solubility in the solvents used, leading to turbidity in the solutions and complicating accurate quantification. Similarly to polyphenols, flavonoid extraction with isopropanol was the least effective, frequently falling below the LOD. Other studies also confirm low levels of flavonoids in invasive plants, with flavonoid content only up to 6.58% of the total phenolic content. Identifying individual compounds remains a challenge: the identification success rate is only up to 56.66% using HPLC [85].

Variability in total polyphenol content due to geographic distribution is not well described, though certain patterns have been observed. For example, in the invasive plant *Ambrosia artemisiifolia*, environmental variables and adaptive strategies in response to heterogeneous environmental conditions across latitudes and longitudes influence seed size and fatty acid content. The plant species studied in this manuscript are widely distributed, with greater abundance in the northern regions, where climatic conditions may affect polyphenol content as a response to ecological and physiological stress. In respect to environmental conditions in our study, only *Impatiens glandulifera* could have circumstances which may affect the production of polyphenols. For *Solidago canadensis*, significant variability in polyphenol content has been reported between China [77,86], Lithuania [78], and Latvia, with the lower concentration in China attributed to the use of more dilute aqueous ethanol for extraction. Lower polyphenol concentrations were found in *Echinocystis lobata* in our results compared to results from Romania, highlighting the complex influences of environmental and climatic factors on the formation of secondary metabolites [40]. Despite these differences, the polyphenol concentrations in the studied plants and their parts are comparable to those in medicinal plants used as dietary polyphenol sources [82,84,87,88].

Our results showed no relation between the content of polyphenols and antioxidant activity, which may depend on differences in the composition of polyphenols as well as other phytochemicals found in plants, such as essential oils and alkaloids [89]. Our study of alkaloid content in *L. polyphyllus* revealed notable quantities, with dominating quinolizidine-type alkaloids. The presence of these alkaloids poses limitations for polyphenol extraction and application without extra purification of the extracts. Interestingly, ethanol proved to be the most effective solvent for both alkaloid and polyphenol extraction.

For antioxidant activity, isopropanol provided better results than for total polyphenols and can be considered a useful solvent for such applications. Methanol and ethanol were similar regarding the obtained results, though ethanol exhibited slightly higher antioxidant activity in *Heracleum sosnowskyi* and *Solidago canadensis.* This variability can be explained by differences in plant phenolic compounds, other phytochemicals in the extracts, and solvent-specific effects [90]. The radical scavenging activity is strongly influenced by the presence of unsaturated long-chain alcohol esters and fatty acids in *Lupinus polyphyllus*, as well as volatile compounds in *Heracleum sosnowskyi*. These major differences in the yields of the studied substances and parameters led to changes in extraction efficiency where methanol and especially ethanol showed higher capacity than isopropanol. Consequently, despite the lower content of polyphenols in *Heracleum sosnowskyi* and *Solidago canadensis*, their biological activity remained higher and comparable to *Impatiens glandulifera.* Consistent with the polyphenol concentrations, flowers and leaves demonstrated the highest biological activity, while stems and roots were more effective in antioxidant assays than in antiradical activity, with *Impatiens glandulifera* displaying particularly high activity.

## 4. Materials and Methods

### 4.1. Plant Material

The plants and their parts were gathered in the period from June to September 2021 in the flowering season. Seeds and fruits were gathered later in the same sampling plots. The altitude of sampling plots did not exceed 30 m above mean sea level. All samples were gathered in Latvia—*Impatiens glandulifera* (coordinates: N 56.992754; E 24.214481); *Lupinus polyphyllus* (N 57.067301; E 24.321872); *Echinocystis lobata* (N 56.848665; E 23.572221); *Heracleum sosnowskyi* (N 56.816618; E 24.245028); *Solidago canadensis* (coordinates: N 56.936525; E 24.094636); *Elodea canadensis* (N 57.125862; E 24.269502)—and identified phenotypically by a botanist. Samples were delivered to the laboratory after harvest (within 3 h of harvesting), where they were dried at 40 °C for 48 h (Gallenkamp Plus II Oven) or until no further change in the sample mass was recorded. Residual raw sample biomass was stored in the freezer (−18 °C) and the samples were dried and prepared for extraction in the same manner as described above. All the results have been expressed as the measured parameter per 100 g of the dried plant biomass—dry weight (DW).

### 4.2. Extraction of Invasive Plant Biomass

For determination of total phenols, flavonoids, and antioxidant and antiradical activity, 50 g of invasive plant parts were homogenized using Polytron (PT 1200E) (Kinematica, Luzern, Switzerland), sieved <1 mm, then 1.5 g of the sieved sample was extracted with 7 mL of solvent using an ultrasound bath (08895-22) (Cole-Parmer, Vernon Hills, IL, USA). After a 30 min extraction at ambient temperature, samples were filtered with a filter paper (Watman no.1). Extraction was replicated 5 times and each solution was diluted to 25 mL. Sample solutions were stored at 4 ± 1 °C until analysed.

### 4.3. Extraction of Lupinus Biomass for Determination of Alkaloid Content

The dried invasive plant biomass was milled using an IKA laboratory-scale blade mill with a 1 mm sieve size to increase the extraction efficiency. Then, 1 g of the milled plant biomass was weighed into 100 mL extraction vessels, and 50 mL of the extraction solvent (70% aqueous ethanol, isopropanol, or methanol) was added. The extraction vessel was then placed in an ultrasonic bath (Cole-Parmer, 300 W) for 15 min, after which the water in the ultrasonic bath was drained and replaced to avoid excessive heating of the samples, and then the samples were sonicated for another 15 min. After sonication, the samples were removed from the ultrasound bath and filtered through Whatman Grade 598 filter paper into a 100 mL volumetric flask. The used filter paper was then placed back into the extraction vessel and another 25 mL of the respective solvent was added for re-extraction of the plant biomass to ensure complete extraction of the compounds of interest. The extraction vessel was then placed in the ultrasound bath for 15 min, after which the samples were filtered as described before and the resulting extracts were combined. The re-extraction process was repeated two times. The resulting extract was diluted up to the 100 mL volumetric flask mark. The resulting extracts were used for the determination of *L. polyphyllus*’s alkaloid content.

### 4.4. Determination of Total Polyphenolics

Total polyphenolic contents were determined using the Folin–Ciocalteu method [91] adjusted for a 96-well microplate reader. A 0.4 M dilution of the Folin–Ciocalteu reagent (Sigma Aldrich, Darmstadt, Germany) was made. A standard solution of gallic acid at a concentration of 1 mg/mL in 96% ethanol and anhydrous Na_2_CO_3_ at a concentration of 10% (*w*/*v*%) was made. Then, 50 µL of deionized water was used to fill each well, and 50 µL of the sample or standard was then added (diluted if necessary). Next, 85 µL of the Folin–Ciocalteu reagent was added and shaken for 5 min during the incubation period. After the incubation, 65 µL of Na_2_CO_3_ was added and incubated for 30 min while shaking. After this incubation, absorbance measurements of the samples and standards were made against reagent blanks (using deionized water as a sample) at 750 nm. The sample concentration was then determined by applying the regression equation to build the calibration curve. The results were then expressed as total polyphenolics g/100 g of dry weight (DW) plant material.

### 4.5. Determination of Total Flavonoids

A modified flavonoid method was used to estimate the total flavonoids in prepared plant extracts [92]. A 10% AlCl_3_ solution and a 1 M potassium acetate solution were prepared. The standard (quercetin) was dissolved in 96% EtOH in the concentration interval of 19–171 µg/mL. LOD (9 µg/mL) and LOQ (30 µg/mL) values were calculated to evaluate the total flavonoid results. A quercetin working solution was prepared by mixing potassium acetate solution, AlCl_3_ solution, 96% ethanol, and demineralized water in a ratio of 1:1:6:11. Then, 50 µL of sample or standard were placed into the wells of the 96-well plate along with 150 µL of the flavonoid working solution and mixed. The prepared samples were then incubated for 30 min, after which the plate was read at 415 nm using an extraction solvent and the flavonoid working solution as the blank. The flavonoid concentration was calculated according to the constructed regression equation and expressed as g QEQ/100 g dried extract (*n* = 5).

### 4.6. Determination of Antioxidative Potential Using DPPH

The radical scavenging potential was calculated using a modified 2,2-Diphenyl-1-picrylhydrazyl (DPPH) antioxidant activity measurement technique [93]. An amount of 24 mg of DPPH was weighed and dissolved in 80% methanol, producing a DPPH stock solution. Then, 50 mL of the stock solution was diluted with 50 mL of 80% methanol to create the DPPH working solution. Trolox at a concentration of 1 mg/mL in ethanol was used as the standard. The 96-well plate received 50 µL of the appropriate sample or standard and 150 µL of the DPPH working solution. The plate was put in the plate reader (Tecan Infinite Pro 200, Männedorf, Switzerland), and the sample was mixed during the pre-incubation time of 10 min (5 min of orbital shaking at 2 mm amplitude). Samples were measured at 517 nm after incubation. The regression equation was used to calculate the concentration, and the results were expressed as mg Trolox equivalent per 100 g dry weight (DW) of plant material.

### 4.7. Determination of Antioxidative Potential Using ABTS

A modified 2,2′-azino-bis(3-ethylbenzothiazoline-6-sulfonic acid antioxidant) potential (ABTS) method was used to estimate the radical scavenging potential [94]. A phosphate buffer (PBS) was prepared by weighing 0.27 g of KH_2_PO_4_, 1.42 g Na_2_HPO_4_, 8.18 g NaCl, and 0.15 g KCl in 1 L of demineralized water. The pH was adjusted to 7.4 with 1 M NaOH. The ABTS stock solution was prepared in the concentration of 2 mM by dissolving 0.1035 g of ABTS in 100 mL PBS buffer. The K_2_S_2_O_8_ solution in the concentration of 70 mM was prepared by weighing out 0.9510 g of K_2_S_2_O_8_ in 50 mL demineralized water. Trolox was used as the standard in the concentration interval of 7–72 µg/mL. LOD (3 µg/mL) and LOQ (11 µg/mL) values were calculated to evaluate the ABTS results. A day before the ABTS measurements, the ABTS working solution was prepared by reacting 50 mL ABTS stock solution with 200 µL K_2_S_2_O_8_ solution. The ABTS working solution was then left in the dark for 16 h for the radical to develop. The next day, the ABTS working solution was diluted to absorbance 1.100 with PBS buffer and used for the measurements. Then, 50 µL of the sample or the standard was transferred into the wells of the 96-well plate with 150 µL ABTS working solution, incubated for 30 min and measured at 734 nm using PBS or a sample solvent with the ABTS working solution as the blank. The antioxidative potential was calculated using the obtained regression equation and expressed as TE g/100 g dried extract (*n* = 5).

### 4.8. Determination of Ferric Reducing Antioxidant Potential Using FRAP

A modified ferric reducing antioxidant potential (FRAP) method was used to estimate the radical scavenging potential [95]. We prepared 0.010 M TPTZ solution, 0.3 M sodium acetate solution at pH 3.6, and 0.02 M FeCl_3_·6H_2_O solutions. The standard (Trolox) was dissolved in 96% ethanol in the concentration interval of 4–46 µg/mL. LOD (2 µg/mL) and LOQ (8 µg/mL) values were calculated to evaluate the FRAP results. The FRAP working solution was prepared on the day of use by mixing sodium acetate buffer, TPTZ, and FeCl_3_·6H_2_O solution in a ratio of 10:1:1. We added 20 µL of sample or standard into the wells of the 96-well plate along with 180 µL of the FRAP reagent. The prepared samples were then incubated for 30 min, after which the plate was read at 593 nm using an extraction solvent and the FRAP reagent as the blank. The FRAP potential was calculated according to the constructed regression equation and expressed as TE g/100 g dried extract (*n* = 5).

### 4.9. Determination of CUPARC

A modified dicopper;2-[1-(4-chlorobenzoyl)-5-methoxy-2-methylindol-3-ylacetate (CUPRAC) method was used to estimate the radical scavenging potential [96]. We prepared 1 M ammonium acetate solution at pH 7.0, 0.75 mM neocuprine solution in 96% EtOH, and 10 mM CuCl_2_ solution. The standard (Trolox) was dissolved in ethanol in the concentration interval of 12–208 µg/mL. LOD (13 µg/mL) and LOQ (45 µg/mL) values were calculated to evaluate the CUPRAC results. The CUPRAC working solution was prepared on the day of use by mixing ammonium acetate buffer, neocuprine, and CuCl_2_ solution in a ratio of 6:5:5. We added 40 µL of sample or standard into the wells of the 96-well plate along with 160 µL of the CUPRAC reagent. The prepared samples were then incubated for 30 min, after which the plate was read at 450 nm using an extraction solvent and the CUPRAC reagent as the blank. The CUPRAC potential was calculated according to the constructed regression equation and expressed as TE g/100 g dried extract (*n* = 5).

### 4.10. Determination of Alkaloid Content in Lupinus polyphyllus

*L. polyphyllus* ethanol, methanol, and isopropanol extracts were used for the determination of qualitative and quantitative alkaloid content using GC-MS. An amount of 50 mL of the obtained extracts was placed in a 100 mL round-bottom flask and evaporated to dryness under reduced pressure (Heidolph RotaVap, Schwabach, Germany). The analysis of alkaloids was carried out in line with the suggestions of previous studies [97]. The resulting dried extract was used for the analysis of lupin alkaloids. Then, 5 mg of the dried extract was placed into a 2 mL GC vial, 1 mL pyridine (>99.99%, Sigma-Aldrich, St. Louis, MO, USA) was added, and the samples were slightly warmed <40 °C to increase the solubility of the extracts. The prepared samples were then placed into the auto sampler of the GC-MS (Shimadzu GC 2010 plus, QP2010, Kyoto, Japan). To prepare the stock solution for quantitative analysis, 10 mg of 99.9% sparteine (C_15_H_26_N_2_) was weighed into a 10 mL volumetric flask and diluted to mark with pyridine (C_5_H_5_N). Standard solutions were prepared by diluting the stock (1 mg sparteine/mL) in a concentration range from 0.01 to 0.85 mg/mL, and the linear standard curve was constructed and used for the calculations.

The following temperature program was used for the analysis of alkaloid extracts: initial temperature 75 °C (hold for 2 min), 20°/min temperature increase to 130 °C and hold for 10 min, and 4 °C/min temperature increase to 310 °C and hold for 10 min. The gas pressure was 77.8 kPa with a total flow of 16.0 mL/min. The injection temperature was 290 °C, and the split ratio 10 with gas flow 3.0 mL/min. A universal Rxi-5MS column (30 m 0.25 × 0.25 µm, Supelco) was used for the analyses. The MS mass/charge ratio scan range was set from 35 *m*/*z* to 650 *m*/*z*. The ion source temperature was 230 °C, the interface temperature 290 °C, and the scanning speed 2500 scans per second. The obtained chromatograms were manually integrated to determine the composition and quantitative amount of the substances present in the extract by determining the mass spectra and peak areas of the recorded signals. Compounds were identified using the NIST 17 integrated mass-spectral database. The amount of alkaloids detected in the samples was expressed as mg sparteine equivalents per 100 g of dry sample.

## 5. Conclusions

The total polyphenol content of six invasive plant species was analysed to better understand their phytochemical composition and to explore the valorisation potential of plant biomass following eradication measures. Among the studied plants, *Impatiens glandulifera* and *Lupinus polyphyllus* were identified as particularly rich in polyphenols, with significant variations in concentration observed across their specific parts. The highest polyphenolic concentrations were found in leaves and flowers, whereas seeds exhibited lower concentrations due to the presence of other compounds, including lipids and fatty acids. In the case of *Lupinus polyphyllus*, the potential advantages and disadvantages of alkaloid content must be considered prior to its utilisation, as these compounds could impose certain limitations. Extracts from the studied plants demonstrated high radical scavenging activity, highlighting the strong oxidative stress tolerance of invasive plant species. These properties suggest potential applications of such extracts. Among the studied plants, *Impatiens glandulifera* exhibited the highest activity; however, other species, such as *Heracleum sosnowskyi* and *Solidago canadensis*, also showed promising potential. Regarding the solvents used in the extraction process, more polar solvents, such as methanol and ethanol, yielded the highest polyphenol yield and antiradical activity. Isopropanol showed smaller differences in comparison to other used solvents in terms of antiradical activity; however, the use of isopropanol resulted in lower flavonoid contents.

This study demonstrates that polyphenols, found in all parts of studied invasive plants, contribute to their stress resistance and play a crucial role in their allelochemical activity. Moreover, the high polyphenol content supports the strategy of invasive plant biomass utilization after eradication (mechanical removal). Specifically, plant biomass can serve as a rich source of polyphenols, with ethanol—as a sustainable and environmentally friendly “green” solvent—being particularly suitable for the extraction.

## Figures and Tables

**Table 1:** Total extraction yield of methanol (MeOH), ethanol (EtOH), and isopropanol (iPrOH) extracts from studied plants and their organs. Results expressed as % of dry matter extracted from the plant biomass.

Plants and Their Parts		MeOH Extract, DW, %	EtOH Extract, DW, %	iPrOH Extract, DW, %
*Impatiens glandulifera*	Leaves	13.26	8.19	8.77
	Stem	14.22	18.50	1.20
	Root	10.92	6.65	0.83
	Seeds	14.35	8.16	13.21
	Flowers	10.78	11.57	6.87
*Lupinus polyphyllus*	Leaves	14.87	17.46	6.79
	Stem	15.24	9.87	12.91
	Root	9.38	14.83	1.39
	Seeds	14.41	12.58	3.02
	Flowers	17.71	10.90	1.26
	Pod	6.64	9.36	0.66
*Echinocystis lobata*	Leaves	12.15	13.85	3.05
	Stem	9.88	16.12	1.29
	Fruit	15.53	11.11	2.29
	Seeds	12.76	8.69	16.56
*Heracleum sosnowskyi*	Leaves	15.02	14.18	2.23
	Stem	14.48	14.98	1.83
	Root	15.07	14.47	3.13
	Flowers	16.63	10.29	2.86
	Seeds	11.69	17.39	23.41
*Elodea canadensis*	Whole plant	3.95	16.26	0.69
*Solidago canadensis*	Leaves	11.00	15.69	2.23
	Stem	6.20	11.35	1.86
	Root	6.83	7.02	2.18
	Flowers	16.52	17.54	1.93

**Table 2:** Total flavonoids (g QEQ/100 g DW) of *Lupinus polyphyllus*, *Impatiens glandulifera*, *Heracleum sosnowskyi*, *Solidago canadensis*, *Echinocystis lobata*, and *Elodea canadensis* using different solvents (MeOH—methanol, EtOH—ethanol, iPrOH—iso-propanol).

Plants and Their Parts		Total Flavonoids, g QEQ/100 g DW		
		MeOH	EtOH	iPrOH
*Impatiens glandulifera*	Leaves	1.53 ± 0.06	0.48 ± 0.05	0.45 ± 0.04
	Stem	0.50 ± 0.05	0.44 ± 0.05	<LOD
	Root	0.53 ± 0.05	0.42 ± 0.05	<LOD
	Seeds	<LOD	<LOD	<LOD
	Flowers	2.28 ± 0.06	1.57 ± 0.03	0.81 ± 0.06
*Lupinus polyphyllus*	Leaves	1.04 ± 0.02	2.19 ± 0.11	1.78 ± 0.06
	Stem	0.45 ± 0.02	0.54 ± 0.02	<LOD
	Root	0.93 ± 0.03	0.63 ± 0.01	<LOD
	Seeds	<LOD	<LOD	<LOD
	Flowers	2.49 ± 0.07	2.43 ± 0.01	0.46 ± 0.02
	Pod	1.37 ± 0.04	1.54 ± 0.03	<LOD
*Echinocystis lobata*	Leaves	0.66 ± 0.02	1.94 ± 0.07	0.59 ± 0.03
	Stem	0.62 ± 0.02	0.70 ± 0.01	<LOD
	Fruit	0.37 ± 0.02	0.73 ± 0.03	0.41 ± 0.02
	Seeds	<LOD	<LOD	<LOD
*Heracleum sosnowskyi*	Leaves	1.24 ± 0.02	2.28 ± 0.02	0.88 ± 0.02
	Stem	0.44 ± 0.03	0.53 ± 0.01	0.38 ± 0.03
	Root	0.61 ± 0.03	0.47 ± 0.03	0.55 ± 0.04
	Flowers	1.86 ± 0.02	2.90 ± 0.05	2.41 ± 0.05
	Seeds	<LOD	<LOD	<LOD
*Elodea canadensis*	Whole plant	0.39 ± 0.02	0.65 ± 0.03	<LOD
*Solidago canadensis*	Leaves	0.72 ± 0.05	0.53 ± 0.05	0.50 ± 0.05
	Stem	<LOD	<LOD	<LOD
	Root	0.41 ± 0.04	0.38 ± 0.04	0.40 ± 0.04
	Flowers	1.13 ± 0.03	1.15 ± 0.03	0.52 ± 0.05

<LOD—below the limit of detection; g QEQ/100 g DW—grams of quercetin equivalents per 100 g of dry plant weight; ± represents the standard deviation, *n* = 5.

**Table 3:** Radical scavenging activity assay (DPPH) (mg Trolox equivalent/100 g DW) of *Lupinus polyphyllus*, *Impatiens glandulifera*, *Heracleum sosnowskyi*, *Solidago canadensis*, *Echinocystis lobata*, and *Elodea canadensis* using different solvents.

Part of Plant	Solvent	*Lupinus* *polyphyllus*	*Impatiens glandulifera*	*Heracleum* *sosnowskyi*	*Echinocystis lobata*	*Solidago canadensis*	*Elodea* *canadensis*
Stem	EtOH	36.60 ± 0.51	257.03 ± 2.41	49.8 ± 2.38	22.97 ± 0.11	37.14 ± 0.39	
	iPrOH	72.39 ± 1.18	279.12 ± 2.98	15.41 ± 0.66	13.52 ± 0.16	38.41 ± 0.55	
	MeOH	45.66 ± 0.67	258.04 ± 2.20	41.63 ± 1.33	36.81 ± 0.15	39.36 ± 0.47	
Root	EtOH	75.37 ± 1.49	180.49 ± 2.55	151.78 ± 1.61		68.50 ± 0.47	
	iPrOH	61.63 ± 0.56	220.74 ± 3.09	183.46 ± 2.60		45.68 ± 0.56	
	MeOH	45.55 ± 0.55	172.11 ± 3.30	154.46 ± 2.04		54.39 ± 0.39	
Leaves	EtOH	79.98 ± 0.73	128.75 ± 1.52	139.19 ± 2.40	69.6 ± 0.37	178.22 ± 0.92	31.90 ± 0.37
	iPrOH	66.85 ± 1.39	130.31 ± 0.94	85.04 ± 4.46	66.19 ± 0.14	106.28 ± 0.93	<LOD
	MeOH	105.3 ± 1.26	129.6 ± 0.96	114.37 ± 9.46	56.38 ± 0.28	133.18 ± 0.76	11.09 ± 0.15
Flowers	EtOH	102.02 ± 0.77	335.85 ± 2.11	276.33 ± 2.42		307.22 ± 1.88	
	iPrOH	73.56 ± 1.54	348.28 ± 2.38	245.44 ± 2.13		178.71 ± 1.67	
	MeOH	120.06 ± 1.52	343.06 ± 2.24	260.6 ± 2.06		283.18 ± 1.54	
Seeds	EtOH	20.9 ± 0.16	34.4 ± 0.63	67.8 ± 0.78	29.80 ± 0.38		
	iPrOH	25.63 ± 0.39	30.00 ± 0.30	45.41 ± 0.56	<LOD		
	MeOH	31.72 ± 0.48	45.21 ± 0.61	64.63 ± 0.63	16.63 ± 0.33		
Pod	EtOH	69.93 ± 0.73		Fruit	74.87 ± 0.64		
	iPrOH	34.93 ± 0.56			66.20 ± 0.97		
	MeOH	74.93 ± 0.68			46.39 ± 0.82		

<LOD—below the limit of detection; ± represents the standard deviation, *n* = 5.

**Table 4:** Ferric-reducing antioxidant power (FRAP) (g Trolox equivalent/100 g DW) of *Lupinus polyphyllus*, *Impatiens glandulifera*, *Heracleum sosnowskyi*, *Solidago canadensis*, *Echinocystis lobata*, and *Elodea canadensis* using different solvents.

Part of Plant	Solvent	*Lupinus* *polyphyllus*	*Impatiens glandulifera*	*Heracleum sosnowskyi*	*Echinocystis lobata*	*Solidago canadensis*	*Elodea* *canadensis*
Stem	EtOH	0.649 ± 0.02	0.661 ± 0.03	0.408 ± 0.02	0.530 ± 0.02	0.665 ± 0.03	
	iPrOH	0.546 ± 0.02	0.381 ± 0.07	0.283 ± 0.02	0.324 ± 0.02	0.379 ± 0.02	
	MeOH	0.620 ± 0.01	0.608 ± 0.08	0.439 ± 0.02	0.592 ± 0.03	0.422 ± 0.01	
Root	EtOH	0.505 ± 0.03	0.778 ± 0.03	0.936 ± 0.02		0.936 ± 0.02	
	iPrOH	0.665 ± 0.03	0.511 ± 0.03	0.746 ± 0.03		0.746 ± 0.03	
	MeOH	0.437 ± 0.01	0.684 ± 0.03	0.902 ± 0.01		0.902 ± 0.01	
Leaves	EtOH	0.631 ± 0.02	0.722 ± 0.02	0.851 ± 0.05	0.748 ± 0.03	0.776 ± 0.02	0.332 ± 0.03
	iPrOH	0.572 ± 0.02	0.729 ± 0.02	0.697 ± 0.03	0.849 ± 0.02	0.748 ± 0.07	<LOD
	MeOH	0.668 ± 0.03	0.728 ± 0.06	0.603 ± 0.05	0.777 ± 0.02	0.722 ± 0.01	0.913 ± 0.07
Flowers	EtOH	0.923 ± 0.02	0.886 ± 0.05	1.521 ± 0.06		1.646 ± 0.04	
	iPrOH	0.498 ± 0.02	0.727 ± 0.06	1.119 ± 0.04		1.234 ± 0.08	
	MeOH	0.816 ± 0.03	1.066 ± 0.12	1.289 ± 0.07		0.906 ± 0.03	
Seeds	EtOH	0.340 ± 0.02	0.641 ± 0.02	0.689 ± 0.02	0.449 ± 0.02		
	iPrOH	0.281 ± 0.01	0.147 ± 0.01	0.295 ± 0.01	<LOD		
	MeOH	0.386 ± 0.02	0.724 ± 0.01	0.628 ± 0.03	0.412 ± 0.03		
Pod	EtOH	0.558 ± 0.03		Fruit	1.035 ± 0.03		
	iPrOH	0.373 ± 0.03			1.045 ± 0.06		
	MeOH	0.522 ± 0.03			1.060 ± 0.05		

<LOD—below the limit of detection; ± represents the standard deviation, *n* = 5.

**Table 5:** Radical scavenging activity assay (using ABTS) (g Trolox equivalent/100 g DW) of *Lupinus polyphyllus*, *Impatiens glandulifera*, *Heracleum sosnowskyi*, *Solidago canadensis*, *Echinocystis lobata*, and *Elodea canadensis* using different solvents.

Part of Plant	Solvent	*Lupinus* *polyphyllus*	*Impatiens glandulifera*	*Heracleum sosnowskyi*	*Echinocystis lobata*	*Solidago* *canadensis*	*Elodea* *canadensis*
Stem	EtOH	1.619 ± 0.03	1.189 ± 0.08	0.860 ± 0.04	0.719 ± 0.02	0.855 ± 0.04	
	iPrOH	1.622 ± 0.06	0.814 ± 0.08	0.436 ± 0.04	0.407 ± 0.02	0.886 ± 0.04	
	MeOH	1.631 ± 0.03	1.414 ± 0.19	0.817 ± 0.05	0.747 ± 0.03	0.865 ± 0.02	
Root	EtOH	0.918 ± 0.02	0.844 ± 0.03	1.881 ± 0.05		1.501 ± 0.05	
	iPrOH	0.844 ± 0.03	0.756 ± 0.05	1.795 ± 0.05		1.199 ± 0.04	
	MeOH	0.937 ± 0.02	0.805 ± 0.05	1.864 ± 0.07		1.489 ± 0.06	
Leaves	EtOH	1.603 ± 0.04	1.322 ± 0.07	1.093 ± 0.04	0.669 ± 0.02	0.828 ± 0.04	0.671 ± 0.03
	iPrOH	1.147 ± 0.04	1.309 ± 0.08	0.957 ± 0.04	0.728 ± 0.03	0.970 ± 0.07	0.820 ± 0.03
	MeOH	1.812 ± 0.05	1.403 ± 0.09	1.065 ± 0.04	0.733 ± 0.01	0.880 ± 0.03	1.368 ± 0.04
Flowers	EtOH	2.455 ± 0.05	1.939 ± 0.05	2.908 ± 0.07		2.919 ± 0.07	
	iPrOH	2.626 ± 0.06	1.657 ± 0.06	2.519 ± 0.06		2.325 ± 0.09	
	MeOH	2.424 ± 0.05	1.815 ± 0.06	2.511 ± 0.06		2.880 ± 0.08	
Seeds	EtOH	0.896 ± 0.03	0.984 ± 0.03	0.885 ± 0.04	0.784 ± 0.03		
	iPrOH	0.812 ± 0.03	0.780 ± 0.07	0.702 ± 0.03	<LOD		
	MeOH	1.158 ± 0.03	0.832 ± 0.05	0.895 ± 0.04	0.689 ± 0.04		
Pod	EtOH	0.856 ± 0.03		Fruit	1.541 ± 0.06		
	iPrOH	0.812 ± 0.03			1.704 ± 0.08		
	MeOH	0.833 ± 0.03			1.233 ± 0.07		

<LOD—below the limit of detection; ± represents the standard deviation, *n* = 5.

**Table 6:** Cupric reducing antioxidant capacity (CUPRAC) (g Trolox equivalent/100 g DW) of *Lupinus polyphyllus*, *Impatiens glandulifera*, *Heracleum sosnowskyi*, *Solidago canadensis*, *Echinocystis lobata*, and *Elodea canadensis* using different solvents.

Part of Plant	Solvent	*Lupinus* *polyphyllus*	*Impatiens glandulifera*	*Heracleum* *sosnowskyi*	*Echinocystis lobata*	*Solidago canadensis*	*Elodea* *canadensis*
Stem	EtOH	1.898 ± 0.05	0.867 ± 0.04	1.658 ± 0.05	1.042 ± 0.03	1.354 ± 0.02	
	iPrOH	1.771 ± 0.03	0.533 ± 0.03	0.954 ± 0.03	1.001 ± 0.07	1.420 ± 0.06	
	MeOH	1.846 ± 0.02	0.648 ± 0.02	1.303 ± 0.02	1.075 ± 0.07	1.265 ± 0.03	
Root	EtOH	2.223 ± 0.02	0.823 ± 0.03	2.852 ± 0.06		2.802 ± 0.09	
	iPrOH	3.302 ± 0.07	0.629 ± 0.03	2.938 ± 0.12		3.830 ± 0.07	
	MeOH	2.209 ± 0.03	0.754 ± 0.03	2.057 ± 0.04		1.990 ± 0.05	
Leaves	EtOH	2.278 ± 0.04	1.415 ± 0.05	2.878 ± 0.07	1.560 ± 0.04	1.711 ± 0.11	0.973 ± 0.02
	iPrOH	1.132 ± 0.05	1.275 ± 0.05	1.655 ± 0.04	1.115 ± 0.05	2.855 ± 0.12	<LOD
	MeOH	2.161 ± 0.04	1.440 ± 0.05	2.609 ± 0.05	1.021 ± 0.06	1.650 ± 0.07	2.077 ± 0.09
Flowers	EtOH	4.165 ± 0.04	1.947 ± 0.04	5.074 ± 0.09		4.361 ± 0.07	
	iPrOH	2.177 ± 0.06	1.776 ± 0.05	3.874 ± 0.06		3.354 ± 0.07	
	MeOH	3.909 ± 0.07	2.573 ± 0.06	4.337 ± 0.06		4.186 ± 0.05	
Seeds	EtOH	1.265 ± 0.03	1.079 ± 0.02	1.818 ± 0.02	1.354 ± 0.07		
	iPrOH	0.884 ± 0.03	0.637 ± 0.05	1.183 ± 0.04	<LOD		
	MeOH	0.908 ± 0.02	0.986 ± 0.04	1.475 ± 0.04	0.891 ± 0.05		
Pod	EtOH	1.872 ± 0.05		Fruit	3.368 ± 0.07		
	iPrOH	1.483 ± 0.04			3.031 ± 0.04		
	MeOH	1.928 ± 0.04			3.999 ± 0.04		

<LOD—below the limit of detection; ± represents the standard deviation, *n* = 5.

**Table 7:** Alkaloid composition (mg/100 g DW) and distribution in extracts of *L. polyphyllus* flowers and seeds based on the used extraction solvent. EtOH—ethanol; iPrOH—isopropanol; MeOH—methanol (Continued).

Part of Plant		Flowers			Seeds		
**RT, min**	Compound/Solvent	EtOH	iPrOH	MeOH	EtOH	iPrOH	MeOH
35.328	Tetrahydrorhombifoline	155.18	179.33	359.47	349.74	24.66	281.78
35.648	Phytol	-	-	94.38	-	-	-
37.469	Unknown 1	-	-	-	128.87	18.01	74.26
38.2	d-Lupanine	-	-	-	1338.50	36.43	975.78
38.307	alfa-Lupanine	132.06	67.78	232.01	-	-	-
40.505	Unknown 2	-	-	-	115.93	-	62.53
41.124	3-beta-Hydroxylupanine	69.70	-	-	99.01	-	57.95
45.269	Unknown 3	-	-	-	1608.57	-	996.47
47.93	Heptcosane	73.30	172.78	141.06	-	17.88	-
50.338	Lupanine	92.50	44.14	129.86	137.47	21.56	83.86
50.705	13-Tigloyloxylupanine	96.59	43.96	130.58	152.80	19.76	88.98
51.552	Nonacosane	76.21	568.68	158.00	-	-	-

**Table 8:** Alkaloid composition (mg/100 g DW) and distribution in extracts of *L. polyphyllus* stem, root, and leaves based on the used extraction solvent. EtOH—ethanol; iPrOH—isopropanol; MeOH—methanol.

Part of Plant		Stem			Roots			Leaves		
RT, min	Compound/Solvent	EtOH	iPrOH	MeOH	EtOH	iPrOH	MeOH	EtOH	iPrOH	MeOH
26.684	Isospartaine	-	-	-	-	-	-	-	-	138.83
35.328	Tetrahydrorhombifoline	113.85	37.82	204.91	87.70	16.60	93.64	380.38	57.82	168.03
35.648	Phytol	-	-	-	-	-	-	230.94	61.11	159.03
38.2	d-Lupanine	132.07	50.28	184.73	182.11	19.26	111.88	-	-	-
38.307	alfa-Lupanine	-	-	121.62	-	-	-	835.52	77.93	587.82
44.386	Hydroxylupanine	-	-		56.29	-	49.59	185.13	-	145.06
47.93	Heptacosane	-	38.53	51.31	-	15.28	38.04	88.67	397.24	-
50.338	Lupanine	59.23	26.45	51.29	-	14.64	-	135.06	37.77	92.08
50.705	13-Tigloyloxylupanine	68.70	29.79	57.27	33.67	15.01	39.01	273.93	47.07	213.98
51.552	Nonacosane	-	192.32	70.27	-	15.46	39.45	-	465.39	120.71

## Data Availability

Data are contained within the article.

## References

[1] Coughlan N.E., Lyne L., Cuthbert R.N., Cunningham E.M., Lucy F.E., Davis E., Caffrey J.M., Dick J.T.A. (2020). In the black: Information and educational potential amongst international databases for invasive alien species designated as of Union Concern. Glob. Ecol. Conserv..

[2] Pérez G., Vilà M., Gallardo B. (2022). Potential impact of four invasive alien plants on the provision of ecosystem services in Europe under present and future climatic scenarios. Ecosyst. Serv..

[3] Huebner C.D., Baskin C.C., Baskin J.M. (2022). Chapter 18—Effects of global climate change on regeneration of invasive plant species from seeds. Plant Regeneration from Seeds.

[4] Baquero R.A., Ayllón D., Nicola G.G. (2021). Are the EU biosecurity legislative frameworks sufficiently effective to prevent biological invasions in the Natura 2000 network?—A case study in Mediterranean Europe. Environ. Sci. Policy.

[5] Dai Z.C., Wan L.Y., Qi S.S., Rutherford S., Ren G.Q., Wan J.S.H., Du D.L. (2020). Synergy among hypotheses in the invasion process of alien plants: A road map within a timeline. Perspect. Plant Ecol. Evol. Syst..

[6] Matisone I., Zumberga A., Lībiete Z., Gerra-Inohosa L., Jansons J. (2018). The impact of forest infrastructure reconstruction on expansion of potentially invasive plant species: First results from a study in Latvia. J. For. Sci..

[7] Leostrin A., Pergl J. (2021). Alien flora in a boreal region of European Russia: An example of Kostroma oblast. Biol. Invasions.

[8] Parks C.G., Radosevich S.R., Endress B.A., Naylor B.J., Anzinger D., Rew L.J., Maxwell B.D., Dwire K.A. (2005). Natural and land-use history of the Northwest mountain ecoregions (USA) in relation to patterns of plant invasions. Perspect. Plant Ecol. Evol. Syst..

[9] Vicente J.R., Pereira H.M., Randin C.F., Gonçalves J., Lomba A., Alves P., Metzger J., Cezar M., Guisan A., Honrado J. (2014). Environment and dispersal paths override life strategies and residence time in determining regional patterns of invasion by alien plants. Perspect. Plant Ecol. Evol. Syst..

[10] Haubrock P.J., Turbelin A.J., Cuthbert R.N., Novoa A., Taylor N.G., Angulo E., Ballesteros-Mejia L., Bodey T.W., Capinha C., Diagne C. (2021). Economic costs of invasive alien species across Europe. NeoBiota.

[11] López-Núñez F.A., Marchante E., Heleno R., Duarte L.N., Palhas J., Impson F., Freitas H., Marchante H. (2021). Establishment, spread and early impacts of the first biocontrol agent against an invasive plant in continental Europe. J. Environ. Manag..

[12] European Commission. https://ec.europa.eu/environment/nature/invasivealien/list/index_en.htm.

[13] Evarts-Bunders P., Evarte-Bundere G. (2020). Development and approbation of methodology for monitoring invasive plant species: The case of Latvia. Thaiszia J. F Bot..

[14] Rutkovska S., Pučkina I., Frolova O. (2017). Inventory of the most invasive alien plant species of Latvia in the “Daugavas loki” Nature Park. Environ. Technol. Resour. Proc. Int. Sci. Pract. Conf..

[15] Sheng Q., Zhao B., Huang M., Wang L., Quan Z., Fang C., Li B., Wu J. (2014). Greenhouse gas emissions following an invasive plant eradication program. Ecol. Eng..

[16] Yoshida K., Hata K., Kawakami K., Hiradate S., Osawa T., Kachi N. (2019). Ecosystem changes following the eradication of invasive species: Evaluation of various eradication scenarios by computer simulation. Ecol. Model..

[17] Van Wilgen B.W., Wannenburgh A., Wilson J.R.U. (2022). A review of two decades of government support for managing alien plant invasions in South Africa. Biol. Conservat..

[18] Sinha A., Nath A., Lahkar B.P., Brahma N., Sarma H.K., Swargowari A. (2022). Understanding the efficacy of different techniques to manage *Chromolaena odorata* L., an Invasive Alien Plant in the sub-Himalayan tall grasslands: Toward grassland recovery. Ecol. Eng..

[19] Nguyen D.T.C., Tran T.V., Nguyen T.T.T., Nguyen D.H., Alhassan M., Lee T. (2023). New frontiers of invasive plants for biosynthesis of nanoparticles towards biomedical applications: A review. Sci. Total Environ..

[20] Parsova V., Jankava A., Berzina M., Palabinska A. (2020). Planning and use of areas infested with invasive plants: Case of Latvia. Curr. Trends Nat. Sci..

[21] Darin G.M.S., Schoenig S., Barney J.N.F., Panetta D., DiTomaso J.M. (2011). WHIPPET: A novel tool for prioritizing invasive plant populations for regional eradication. J. Environ. Manag..

[22] Tuck C.O., Perez E., Horvath I.T., Sheldon R.A., Poliakoff M. (2012). Valorization of Biomass: Deriving More Value from Waste. Science.

[23] Feng Q., Wang B., Chen M., Wu P., Lee X., Xing Y. (2021). Invasive plants as potential sustainable feedstocks for biochar production and multiple applications: A review. Resour. Conservat. Recycl..

[24] Williams V.L., Wojtasik E.M., Byrne M.J. (2021). A chronicle of alien medicinal plants used as traditional medicine in South Africa, and their status as invasive species. S. Afr. J. Bot..

[25] Prabakaran K., Li J., Anandkumar A., Leng Z., Zou C.B., Du D. (2019). Managing environmental contamination through phytoremediation by invasive plants: A review. Ecol. Eng..

[26] Pereyra P.J. (2016). Revisiting the use of the invasive species concept: An empirical approach. Austral Ecol..

[27] Shamprasad B.R., Subramani R., Subramaniam S., Sivasubramanian A. (2022). Environmentally benign, ultrasonication assisted, sustainable valorization for commercially important nutraceutical-Daucosterol from the heartwood of invasive *Prosopis juliflora* (Sw.) DC. Sustain. Chem. Pharm..

[28] Ahmed A., Abu Bakar M.S., Hamdani R., Park Y.o.K., Lam S.S., Sukri R.S., Hussain M., Majeed K., Phusunti N., Jamil F. (2020). Valorization of underutilized waste biomass from invasive species to produce biochar for energy and other value-added applications. Environ. Res..

[29] Ganguly R.K., Al-Helal M.d.A., Chakraborty S.K. (2022). Management of invasive weed *Chromolaena odorata* (Siam weed) through vermicomposting: An eco-approach utilizing organic biomass valorization. Environ. Technol. Innov..

[30] Kauser H., Pal S., Haq I., Khwairakpam M. (2020). Evaluation of rotary drum composting for the management of invasive weed Mikania micrantha Kunth and its toxicity assessment. Biores. Technol..

[31] Bandara W.A.R.T.W., Ranasinghe O., Perera P., Vlosky R., Kizha A.R. (2022). Potential to use invasive plants in biomass energy production: A case study *Prosopis juliflora* in coastal wetlands of Sri Lanka. Trees For. People.

[32] Niedrite E., Klavins L., Dobkevica L., Purmalis O., Ievinsh G., Klavins M. (2024). Sustainable control of invasive plants: Compost production, quality, and effects on wheat germination. J. Environ. Manag..

[33] De Lange W.J., Stafford W.H.L., Forsyth G.G., Le Maitre D.C. (2012). Incorporating stakeholder preferences in the selection of technologies for using invasive alien plants as a bio-energy feedstock: Applying the analytical hierarchy process. J. Environ. Manag..

[34] Wolf S., Romeis J., Collatz J. (2018). Utilization of plant-derived food sources from annual flower strips by the invasive harlequin ladybird *Harmonia axyridis*. Biol. Control.

[35] Lin T., Vrieling K., Laplanche D., Klinkhamer P.G.L., Lou Y., Bekooy L., Degen T., Bustos-Segura C., Turlings T.C.J., Desurmont G.A. (2021). Evolutionary changes in an invasive plant support the defensive role of plant volatiles. Curr. Biol..

[36] Luca S.V., Kulinowski Ł., Ciobanu C., Zengin G., Czerwińska M.E., Granica S., Xiao J., Skalicka-Woźniak K., Trifan A. (2022). Phytochemical and multi-biological characterization of two *Cynara scolymus* L. varieties: A glance into their potential large scale cultivation and valorization as bio-functional ingredients. Ind. Crops Prod..

[37] Peter A., Žlabur J.Š., Šurić J., Voća S., Purgar D.D., Pezo L., Voća N. (2021). Invasive Plant Species Biomass—Evaluation of Functional Value. Molecules.

[38] Li Q., Mei H., Zhang Z., Jiang H., Zhang W. (2022). Valorization of flavonoid-rich invasive weed—*Eupatorium adenophorum* in the cleaner production of coloristic and functional wool fabric with a research focus on photofading mechanism. Ind. Crops Prod..

[39] Flórez-Fernández N., Illera M., Sánchez M., Lodeiro P., Torres M.D., López-Mosquera M.E., Soto M., De Vicente M.S., Domínguez H. (2021). Integrated valorization of *Sargassum muticum* in biorefineries. Chem. Eng. J..

[40] Han C., Shao H., Zhou S., Mei Y., Cheng Z., Huang L., Lv G. (2021). Chemical composition and phytotoxicity of essential oil from invasive plant, *Ambrosia artemisiifolia* L. Ecotox. Environ. Saf..

[41] Adelipour M., Cheraghzadeh M., Rashidi M. (2022). Polyphenols as epigenetic modulators in treating or preventing of cancers. Gene Rep..

[42] Klavins M., Purmalis O., Klavina L., Niedrite E., Ansone-Bertina L. (2024). Biomass of Invasive Plants as a Resource for the Development of the Bioeconomy. BioResources.

[43] Duthie G., Gardner P., Kyle J. (2003). Plant polyphenols: Are they the new magic bullet?. Proc. Nutr. Soc..

[44] Khoddami A., Khoddami A., Wilkes M.A., Roberts T.H. (2013). Techniques for analysis of plant phenolic compounds. Molecules.

[45] De Elguea-Culebras G.O., Bravo E.M., Sánchez-Vioque R. (2022). Potential sources and methodologies for the recovery of phenolic compounds from distillation residues of Mediterranean aromatic plants. An approach to the valuation of by-products of the essential oil market—A review. Ind. Crops Prod..

[46] Martins C.C., Rodrigues R.C., Mercali G.D., Rodrigues E. (2022). New insights into non-extractable phenolic compounds analysis. Food Res. Internat..

[47] Rauf A., Nengroo Z. (2020). Fatty acid composition, functional group analysis and antioxidant activity of *Nymphia alba* and *Lupinus polyphyllus* seed extracts. J. Oleo Sci..

[48] Giacomini D., Musumeci R., Galletti P., Martelli G., Assennato L., Sacchetti G., Guerrini A., Calaresu E., Martinelli M., Cocuzza C. (2017). 4-Alkyliden-azetidinones modified with plant derived polyphenols: Antibacterial and antioxidant properties. Eur. J. Med. Chem..

[49] Arnoldi A., Boschin G., Zanoni C. (2015). The health benefits of sweet lupin seed flours and isolated proteins. J. Funct. Foods.

[50] Mikulic-Petkovsek M., Veberic R., Hudina M., Misic E. (2022). HPLC-DAD-MS Identification and Quantification of Phenolic Components in Japanese Knotweed and American Pokeweed Extracts and Their Phytotoxic Effect on Seed Germination. Plants.

[51] Lazzaro L., Essl F., Lugliè A., Padedda B.M., Pyšek P., Brundu G., Mazza G., Tricario E. (2018). Invasive alien plant impacts on human health and well-being. Invasive Species and Human Health.

[52] Kumar R.P., Singh J.S. (2020). Invasive alien plant species: Their impact on environment, ecosystem services and human health. Ecol. Indic..

[53] Ghendov V., Izverscaia T., Şabanova G. (2010). Heracleum sosnowskyi Manden. (*Apiaceae*)—An invasive alien plant for the flora of Republic of Moldova. Horticultură, Viticultură şi Vinificaţie, Silvicultură şi Grădini Publice, Protecţia Plantelor.

[54] Petterson D.S., Colin W., Harold C., Koushik S., Jon F. (2016). Lupin: Overview. Encyclopedia of Food Grains.

[55] Erdemoglu N., Ozkan S., Tosun F. (2007). Alkaloid profile and antimicrobial activity of *Lupinus angustifolius* L. alkaloid extract. Phytochem. Rev..

[56] Romeo F.V., Fabroni S., Ballistreri G., Muccilli S., Spina A., Rapisarda P. (2018). Characterization and antimicrobial activity of alkaloid extracts from seeds of different genotypes of *Lupinus* spp. Sustainability.

[57] Osorio C.E., Till B.J. (2022). A Bitter-Sweet Story: Unraveling the Genes Involved in Quinolizidine Alkaloid Synthesis in *Lupinus albus*. Front. Plant Sci..

[58] Leck M.A., Parker V.T., Simpson R.L. (1989). Ecology of Soil Seed Banks.

[59] Flegel M., Schrader S., Zhang H. (1998). Influence of food quality on the physical and chemical properties of detritivorous earthworm casts. Appl. Soil Ecol..

[60] Hosakatte N.M., Yaser H.D., Dayanand D., Nasser A.S. (2022). Comparative physicochemical analysis of seed oils of wild cucumber (*Cucumis sativus* var. *hardwickii* (Royle) Alef.), cucumber (*Cucumis sativus* L. var. *sativus*), and gherkin (*Cucumis anguria* L.). S. Afr. J. Bot..

[61] Eleomar O.P., Cristina C., Carolina C.G., Isabel C.F.R., Ferreira L.B. (2021). Current status of genus *Impatiens*: Bioactive compounds and natural pigments with health benefits. Trends Food Sci. Technol..

[62] Borek S., Ratajczak W., Ratajczak L. (2015). Regulation of storage lipid metabolism in developing and germinating lupin (*Lupinus* spp.) seeds. Acta Physiol. Plant.

[63] Kviesis J., Kļimenkovs I., Arbidans L., Podjava A., Kļaviņš M., Liepiņš E. (2019). Evaluation of furanocoumarins from seeds of the wild parsnip (*Pastinaca sativa* L. s.l.). J. Chromatogr. B.

[64] Grzędzicka E. (2022). Invasion of the Giant Hogweed and the Sosnowsky’s Hogweed as a Multidisciplinary Problem with Unknown Future—A Review. Earth.

[65] Rodge S., Biradar S.D. (2017). Antioxidant activity of some medicinal plants of family *Cucurbitaceae*. Worlds J. Pharm. Res..

[66] Pulido R., Bravo L., Saura-Calixto F. (2000). Antioxidant activity of dietary polyphenols as determined by a modified ferric reducing/antioxidant power assay. J. Agric. Food Chem..

[67] De Cortes Sánchez M., Altares P., Pedrosa M.M., Burbano C., Cuadrado C., Goyoaga C., Dávila-Ortiz G. (2005). Alkaloid variation during germination in different lupin species. Food Chem..

[68] Van Meerbeek K., Appels L., Dewil R., Calmeyn A., Lemmens P., Muys B., Hermy M. (2015). Biomass of invasive plant species as a potential feedstock for bioenergy production. Biofuels Bioprod. Biorefining.

[69] Linder C.R. (2000). Adaptive Evolution of Seed Oils in Plants: Accounting for the Biogeographic Distribution of Saturated and Unsaturated Fatty Acids in Seed Oils. Am. Nat..

[70] Navarro S.L.B., Capellini M.C., Aracava K.K., Rodrigues C.E.C. (2000). Corn germ-bran oils extracted with alcoholic solvents: Extraction yield, oil composition and evaluation of protein solubility of defatted meal. Food Bioprod. Proc..

[71] Szewczyk K., Zidorn C., Biernasiuk A., Komsta Ł., Granica S. (2016). Polyphenols from *Impatiens* (*Balsaminaceae*) and their antioxidant and antimicrobial activities. Ind. Crops Prod..

[72] Boinik V.V., Akritidu K.P., Demeshko O.V. (2015). Phenolic compounds from roots of *Lupinus polyphyllus*. Chem. Nat. Compd..

[73] Uysal A., Ozer O.Y., Zengin G., Stefanucci A., Mollica A., Picot-Allain C.M.N., Mahomoodally M.F. (2019). Multifunctional approaches to provide potential pharmacophores for the pharmacy shelf: *Heracleum sphondylium* L. subsp. *Ternatum* (Velen.) Brummitt. Comp. Biol. Chem..

[74] Rysiak A., Dresler S., Hanaka A., Hawrylak-Nowak B., Strzemski M., Kováčik J., Sowa I., Latalski M., Wójciak M. (2021). High Temperature Alters Secondary Metabolites and Photosynthetic Efficiency in *Heracleum sosnowskyi*. Int. J. Mol. Sci..

[75] Bahadori M.B., Dinparast L., Zengin G. (2016). The Genus *Heracleum*: A Comprehensive Review on Its Phytochemistry, Pharmacology, and Ethnobotanical Values as a Useful Herb. Comprehen. Rev. Food Sci. Food Saf..

[76] Weryszko-Chmielewska E., Chwil M. (2017). Localisation of furanocoumarins in the tissues and on the surface of shoots of *Heracleum Sosnowskyi*. Bot..

[77] Deng Y., Zhao Y., Padilla-Zakour O., Yang G. (2015). Polyphenols, antioxidant and antimicrobial activities of leaf and bark extracts of *Solidago canadensis* L. Ind. Crops Prod..

[78] Radusiene J., Marska M., Ivanauskas L., Jakstas V., Karpaviciene B. (2015). Assessment of phenolic compound accumulation in two widespread goldenrods. Ind. Crops Prod..

[79] Dogan M., Saygideger S.D., Colak U. (2009). Effect of Lead Toxicity on Aquatic Macrophyte *Elodea canadensis* Michx. Bull Environ. Contam Toxicol.

[80] Mues R. (1989). Species Specific Flavone Glucuronides in Elodea Species. Biochem. Syst. Ecol..

[81] Erhard D., Gross E.M. (2006). Allelopathic activity of *Elodea canadensis* and *Elodea nuttallii* against epiphytes and phytoplankton. Aq. Bot..

[82] Ielciu I., Hanganu D., Păltinean R., Vlase L., Frédérich M., Gheldiu A.M., Benedec D., Crişan G. (2018). Antioxidant capacity and polyphenolic content of the *Echinocystis lobata* (Michx.) Torr. Et A.Gray flowers. Pak. J. Pharm. Sci..

[83] Li Z.H., Wang Q., Ruan X., Pan C.D., Jiang D.A. (2010). Phenolics and plant allelopathy. Molecules.

[84] Vinogradova Y., Shelepova O., Vergun O., Grygorieva O., Brindza J. (2021). Phenolic content and antioxidant activity of *Echinocystis lobata* (Mich.) Torr. ET Gray (*Cucurbitaceae*). Potravin. Slovak J. Food Sci..

[85] Cucu A.A., Baci G.M., Cucu A.B., Dezsi S., Lujerdean C., Hegedus I.C., Bobis O., Moise A.R., Dezmirean D.S. (2022). *Calluna vulgaris* as a Valuable Source of Bioactive Compounds: Exploring Its Phytochemical Profile, Biological Activities and Apitherapeutic Potential. Plants.

[86] Kato-N H., Kato M. (2022). Allelopathy and Allelochemicals of *Solidago canadensis* L. and *S. altissima* L. for Their Naturalization. Plants.

[87] Katalinic V., Milos M., Kulisic T., Jukic M. (2006). Screening of 70 medicinal plant extracts for antioxidant capacity and total phenols. Food Chem..

[88] Vinson J.A., Su X., Zubik L., Bose P. (2001). Phenol antioxidant quantity and quality in foods: Fruits. J. Agric. Food Chem..

[89] Bernal F.A., Coy-Barrera E. (2022). Composition and Antifungal Activity of the Alkaloidal Fraction of *Lupinus mirabilis* Leaves: A Biochemometrics-Based Exploration. Molecules.

[90] Saito S., Okamato Y., Kawabata J. (2004). Effects of Alcoholic Solvents on Antiradical Abilities of Protocatechuic Acid and Its Alkyl Esters. Biosci. Biotechnol. Biochem..

[91] Attard E. (2013). A rapid icrotiter plate *Folin-Ciocalteu* method for the assessment of polyphenols. Open Life Sci..

[92] Zhang C., Shen Y., Chen J., Xiao P., Bao J. (2008). Nondestructive prediction of total phenolics, flavonoid contents, and antioxidant capacity of rice grain using near-infrared spectroscopy. J. Agric. Food Chem..

[93] Fukumoto L.R., Mazza G. (2000). Assessing antioxidant and prooxidant activities of phenolic compounds. J. Agric. Food Chem..

[94] Granados-Guzmán G., Salazar-Aranda R., Garza-Tapia M., Castro-Ríos R., Waksman de Torres N. (2017). Optimization, and validation of two high-throughput methods indicating antiradical activity. Curr. Anal. Chem..

[95] Klavins L., Perkons I., Mezulis M., Viksna A., Klavins M. (2022). Procyanidins from cranberry press residues—Extraction optimization, purification, and characterization. Plants.

[96] Khursheed A., Ahmad S., Saleem M., Khan K.U.R., Khan J., Orhan I.E., Khurshid U. (2022). Phytochemical profiling, in vitro biological activity, docking studies, and cytotoxicity assessments of *Rondeletia odorata* Jacquin: An unexplored plant of the coffee family. Front. Chem..

[97] Cely-Veloza W., Quiroga D., Coy-Barrera E. (2022). Quinolizidine-based variations and antifungal activity of eight *Lupinus* species grown under greenhouse conditions. Molecules.

